# Complement-mediated thrombotic microangiopathy secondary to sepsis-induced disseminated intravascular coagulation successfully treated with eculizumab: A case report

**DOI:** 10.1097/MD.0000000000006056

**Published:** 2017-02-10

**Authors:** Tomohiro Abe, Akira Sasaki, Taichiro Ueda, Yoshitaka Miyakawa, Hidenobu Ochiai

**Affiliations:** a Department of Trauma and Critical Care Medicine, University of Miyazaki Hospital, Miyazaki, Japan; b Department of General Internal Medicine, Saitama Medical University, Saitama, Japan.

**Keywords:** complement activation, complement inactivating agents, disseminated intravascular coagulation, sepsis, systemic inflammatory response syndrome, thrombotic microangiopathies

## Abstract

Secondary thrombotic microangiopathies (TMAs) are induced by several underlying conditions; most are resolved by treating background disease. Eculizumab is a human monoclonal antibody that blocks the final stage of the complement system and effectively treats atypical hemolytic uremic syndrome (aHUS). In this report, we present a patient with TMA secondary to sepsis- induced coagulopathy, who was successfully treated with eculizumab.

A 44-year-old woman, who had no special medical history or familial history of TMAs, was admitted on suspicion of septic shock. Physical examination revealed gangrene on her soles. Blood tests revealed a decreased platelet count, disseminated intravascular coagulation (DIC), renal dysfunction, hemolysis, and infection. Although the coagulation disorder improved with intensive care, the low platelet count, elevated lactate dehydrogenase levels, and renal dysfunction persisted. Our investigations subsequently excluded thrombotic thrombocytopenic purpura and Shiga toxin-producing Escherichia coli-induced HUS. Plasma exchange only improved lactate dehydrogenase levels. We clinically diagnosed this case as atypical HUS and started eculizumab treatment. The patient's platelet count increased, her renal dysfunction improved, and the gangrene on her feet was ameliorated. The patient was discharged without maintenance dialysis therapy after approximately 3 months. Subsequent tests revealed elevated serum levels of soluble C5b-9, and genetic testing revealed compound heterozygous c.184G > A (Val62Ile) and c.1204T > C (Tyr402His) single-nucleotide polymorphisms in complement factor H.

We encountered a case of complement-mediated TMA accompanied by DIC, which was successfully treated with eculizumab. Further studies are necessary to support the optimal use of eculizumab for TMA with background diseases.

## 1 Introduction

Thrombotic microangiopathies (TMAs) are syndromes characterized by hemolytic anemia, renal dysfunction, and thrombocytopenia, and they are classified as primary or secondary TMAs based on the cause.^[1]^ Secondary TMAs are mainly treated by controlling underlying conditions and by supportive care.^[1,2]^ Eculizumab is a human monoclonal antibody that directly blocks C5 as well as the final stage of complement activation; it is used for treating atypical hemolytic uremic syndrome (aHUS) caused by defective complement control.^[1–5]^ In contrast, disseminated intravascular coagulation (DIC) often arises as a complication of sepsis and other conditions^[6]^ and sometimes induces TMA, especially in severe cases.^[7,8]^ The efficacy and safety of eculizumab for complement-mediated TMA accompanied by DIC are unclear. We herein report a case of complement-mediated TMA with DIC, in which early treatment using eculizumab successfully resolved the TMA.

## 2 Case presentation

A 44-year-old healthy woman was examined at her local medical clinic after developing fever, diarrhea, and vomiting in June 2014. She did not have any personal or family history of renal impairment or pregnancy disorders. She did not receive any medications at home. Examination revealed a significant fever (40.2°C), decreased systolic blood pressure, elevated C-reactive protein (CRP) level (2286 nmol/L; normal range <13.3 nmol/L), and a significantly decreased platelet count (25 × 10^9^/L; normal range 158–348 × 10^9^/L). Based on suspicion of septic shock, the patient was transferred to our facility after treatment with intravenous fluids and 1 g of cefotiam.

**Figure 1:**
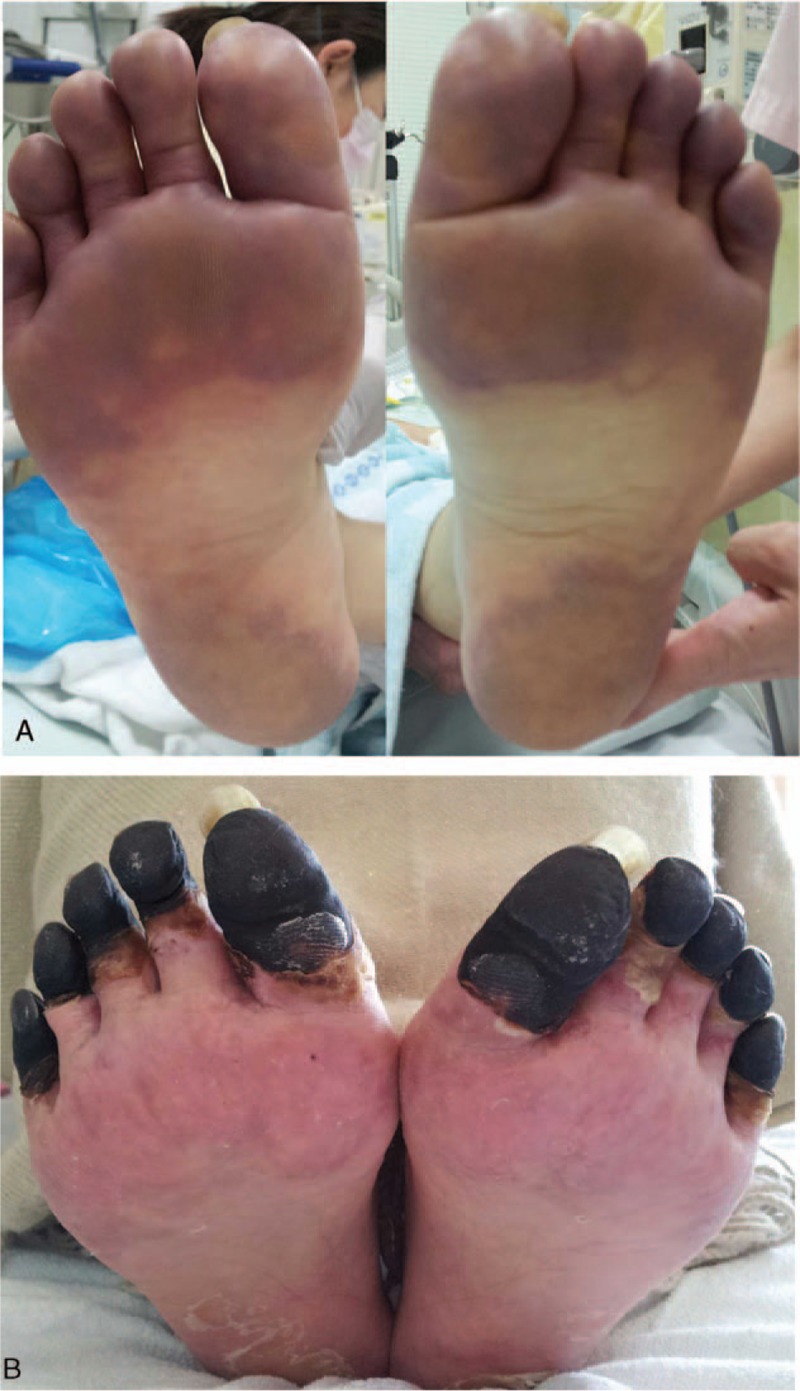
Photographs of the patient's soles. (A) At admission, the patient's soles exhibited purpura that extended to the tips, with tenderness; the purpura worsened before the eculizumab treatment. (B) After the eculizumab treatment, the lesions gradually reduced until only the toes were involved, although a toe amputation was ultimately required.

The patient's vital signs reflected a state of shock: blood pressure was 66/44 mm Hg (with continuous intravenous administration of norepinephrine at 0.17 μg/kg/min), and heart rate was 130 beats/min. No pronounced respiratory disorder was observed. The patient's body temperature had decreased to 37.2°C. Physical examination revealed no clear abnormalities in the trunk and abdomen, although purpura extended along the soles of both feet to the toe tips, with accompanying tenderness (Fig. 1A). A bleeding tendency was suspected, because of sustained bleeding from a blood vessel puncture.

Blood tests revealed a hemoglobin level of 113 g/L (normal range 116–148 g/L) and a substantially decreased platelet count (11 × 10^9^/L). Biochemical tests revealed the following: serum urea nitrogen, 9.96 mmol/L (normal range 2.86–7.14 mmol/L); serum creatinine, 304 μmol/L (normal range 40.7–69.8 μmol/L); total bilirubin, 48 μmol/L (normal range 6.8–26.3 μmol/L); aspartate aminotransferase, 1.92 μkat/L (normal range 0.22–0.5 μkat/L); alanine aminotransferase, 0.57 μkat/L (normal range 0.12–0.38 μkat/L); and lactate dehydrogenase (LDH), 19.1 μkat/L (normal range 2.1–3.7 μkat/L). An elevated CRP level of 1767 nmol/L and a procalcitonin level of 50.3 μg/L (normal range <0.5 μg/L) indicated a pronounced inflammatory response. Coagulation tests revealed the following: prothrombin time (PT), 23.9 s (normal range 10.5–12.5 s) (PT-international normalized ratio [PT-INR]: 3.54); activated partial thromboplastin time (APTT), 101 s (normal range 24–36 s); fibrinogen, 0.72 g/L (normal range 2–4 g/L); D-dimer, 874 nmol/L (normal range <5.5 nmol/L); and fibrin degradation product, 315 mg/L (normal range 1–10 mg/L). These findings confirmed that the patient had DIC concurrent with sepsis. Complement tests revealed that the level of complement component 3 was 0.67 g/L (normal range 0.73–1.38 g/L) and that of complement component 4 was 0.24 g/L (normal range 0.11–0.31 g/L) (Table 1). Total complement activity (CH50) could not be measured because of the test delay.

**Table 1:**
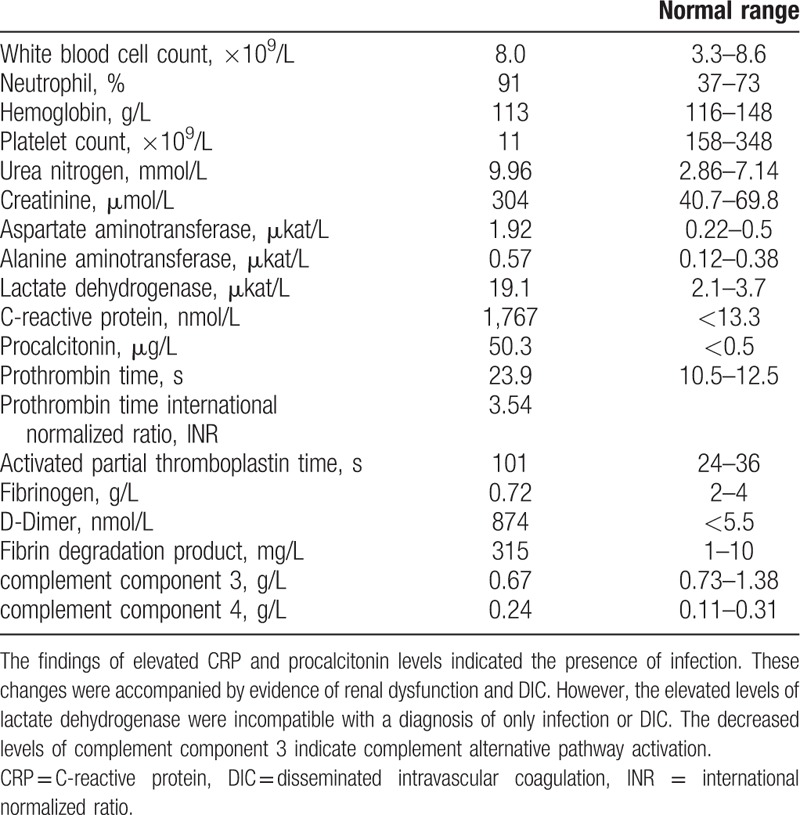
Laboratory values at admission.

Plain chest and abdominal radiography and full-body computed tomography did not reveal findings suggesting the origin of the sepsis-causing infection. On day 1, we initiated treatment using antibacterial agents (meropenem: 2 g/day, linezolid: 1200 mg/day, and clindamycin: 2400 mg/day), noradrenaline, hydrocortisone succinate, and a rapid fluid infusion. We also administered a transfusion of fresh frozen plasma (5 units) and platelet concentrate (10 units) to treat the bleeding tendency. We did not administer recombinant human soluble thrombomodulin or antithrombin III. However, the patient's respiratory condition steadily deteriorated, and we commenced mechanical ventilation.

We received the blood endotoxin test results on day 2, which revealed an elevated endotoxin level of 0.293 EU/mL (normal range <0.05 EU/mL), suggesting a potential gram-negative bacterial infection. Thus, we performed direct hemoperfusion using a polymyxin B-immobilized fiber column. In addition, we started continuous hemodiafiltration (CHDF) because of prolonged anuria. Another blood test revealed decreased serum haptoglobin (type 2-1: 240 mg/L [normal range 380–1790 mg/L]), and schizocytes were observed during microscopic examination of a hemogram. These findings, combined with an elevated LDH level, led us to suspect hemolysis with potential concurrent TMA. However, the patient had a disintegrin-like and metalloproteinase with thrombospondin type 1 motifs, member 13 (ADAMTS13) levels of 41.7% (normal range 70–120%) and tested negative for ADAMTS13 inhibitors and thrombotic thrombocytopenic purpura.

As her serum PT and APTT values improved by day 6 after admission, we initiated heparin treatment for thrombus prevention. Defecation was observed on day 7, which allowed a fecal culture test, although the results were negative for enterohemorrhagic *Escherichia coli*. Immunological test results were negative for O157 lipopolysaccharide, antinuclear, anti-cardiolipin immunoglobulin G (IgG), anti-cardiolipin β2-glycoproteinI complex antibody, myeloperoxidase-anti-neutrophil cytoplasmic, anti-neutrophil cytoplasmic, and anti-smooth muscle antibodies; anti-ds-DNA IgG and immunoglobulin M; and urinary *Streptococcus pneumoniae* antigen. A blood culture did not reveal any bacteria.

On day 8, the patient exhibited improved coagulation function (PT-INR, 1.06; fibrinogen, 2.86 g/L; and APTT, 42.9 s [while receiving heparin at 5,000 U/day]) except for elevated levels of D-dimer at 489.6 nmol/L. However, she exhibited signs of TMA (platelet count, 10 × 10^9^/L; creatinine, 218 μmol/L; LDH, 20.7 μkat/L; and urine volume, ∼100 mL/day) with exacerbated purpura on the soles of her feet.

Although treatment for sepsis improved her DIC, platelet counts remained low, LDH levels remained high, and she exhibited signs of renal dysfunction with anuria and exacerbated purpura on the soles of her feet. Therefore, we started plasma exchange therapy for 4 consecutive days on day 9. Although the LDH level decreased to 4.7 μkat/L 4 days after plasma exchange, the low platelet count persisted (12 × 10^9^/L), and we observed further progression of renal dysfunction (serum creatinine, 290 μmol/L; daily urine volume, <150 mL). Therefore, we treated the patient using intravenous eculizumab (900 mg once a week for 4 weeks) because we clinically diagnosed the case as complement-mediated aHUS.

**Figure 2:**
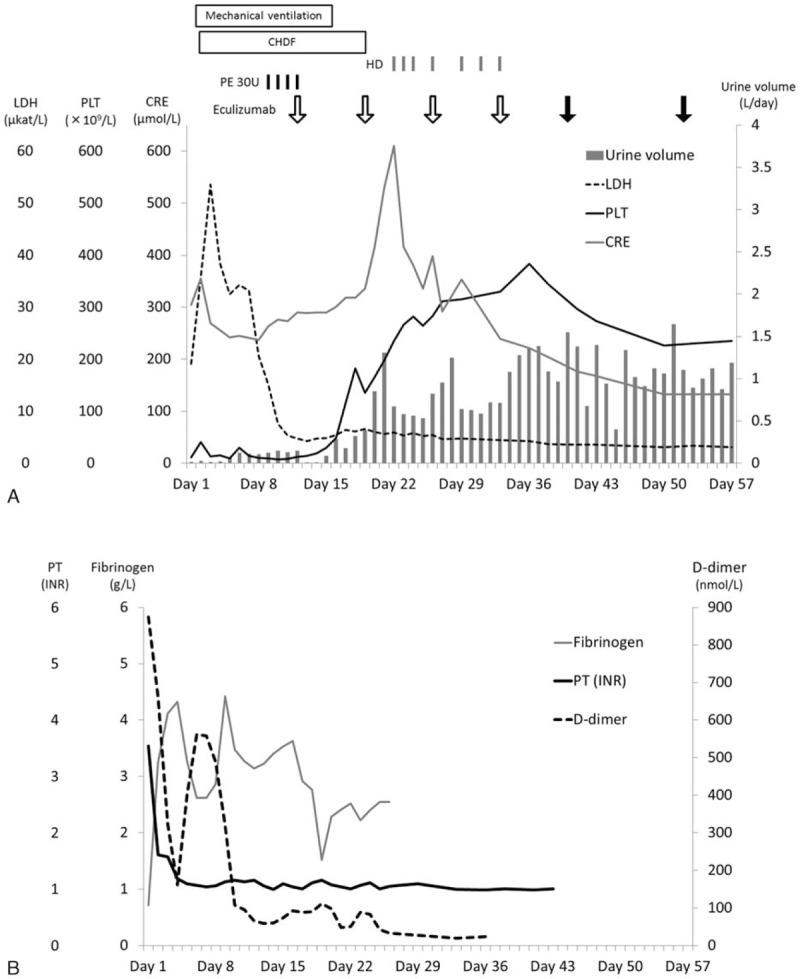
Summary of clinical values of thrombotic microangiopathy (A) and disseminated intravascular coagulation (B). (A) At admission, the patient exhibited symptoms of thrombotic microangiopathy (thrombocytopenia and elevated serum levels of lactate dehydrogenase and creatinine). We clinically diagnosed atypical hemolytic uremic syndrome and performed plasma exchange on day 9. Plasma exchange (black bar) was initiated owing to decreased platelet count (black line) and renal dysfunction (serum creatinine, gray line; urine volume, gray column), which persisted despite decreased lactate dehydrogenase levels (dotted line). We administrated 900 mg of eculizumab (open arrow) on day 12. After eculizumab treatment, the thrombocytopenia and oliguria resolved, although creatinine levels transiently elevated due to transition from continuous hemodiafiltration to intermittent hemodialysis. On day 19, mechanical ventilation was discontinued. Because of an increase of urine volume, continuous hemodiafiltration was discontinued on day 19, and intermitted hemodialysis was performed from day 22 to day 33. The eculizumab dosage was increased to 1200 mg (closed arrow) after the fifth eculizumab administration. Every value of thrombotic microangiopathy remained in remission. (B) The coagulation tests indicated disseminated intravascular coagulation (prolonged prothrombin time, reduced levels of fibrinogen, and elevated levels of D-dimer). The abnormalities indicative of DIC (prothrombin time, black line; fibrinogen, gray line) had resolved except for elevated D-dimer levels (dotted line) on day 8. D-dimer levels normalized after administration of eculizumab. APTT = activated partial thromboplastin time, CHDF = continuous hemodiafiltration, CRE = creatinine, DIC = disseminated intravascular coagulation, HD = hemodialysis, LDH = lactate dehydrogenase, PE = plasma exchange, PLT = platelet count, PT (INR) = prothrombin time international normalized ratio.

On day 15, 4 days after eculizumab treatment, the patient's respiratory condition improved, and we discontinued mechanical ventilation. On day 18, we observed increased urine output (320 mL/day) and a normalized platelet count (182 × 10^9^/L), although there was no marked change in the patient's serum creatinine level (292–318 μmol/L). After the second dose of eculizumab on day 19, the patient exhibited stable urine volumes (500–1,300 mL/day), and we switched her treatment from CHDF to intermittent hemodialysis on day 20. There was a transition to intermittent hemodialysis; therefore, a peak in creatinine levels of 610 μmol/L was observed on day 22, although it subsequently decreased, and the patient discontinued hemodialysis on day 33 (Fig. 2). In addition, we observed that following eculizumab treatment, the purpura had subsided from the soles of her feet and only involved the toes. Therefore, we continued the intravenous eculizumab treatment (1200 mg every 2 weeks from the fifth dose, on day 40) according to the prescribed regimen. For prophylaxis against meningococcal infection, we vaccinated the patient against meningococcus on day 45, and we administered antibiotics (ceftriaxone 1 g/day on days 16–48 and oral amoxicillin 750 mg/day on days 49–60). Consequently, we did not note any further symptom relapse or complications; the patient was ultimately discharged in an ambulatory condition on day 83 without any prescriptions for home medications. Unfortunately, the persistent purpura on her toes consequently necrotized (Fig. 1B) and necessitated a toe amputation, although walking function was preserved. The patient received intravenous eculizumab (1200 mg once every 2 weeks) on an outpatient basis. The patient did not experience TMA symptoms or infections including meningococcal infections. Finally, we discontinued eculizumab administration 22 months since the onset of disease. After discontinuation of eculizumab, she has not shown signs of TMA.

Subsequent testing revealed elevated serum soluble C5b-9 levels of 870 ng/mL (normal control, 336.2 ng/mL), indicating abnormal complement activation. Anti-complement factor H (CFH) antibodies were absent. Genetic analysis revealed c.184G>A (Val62Ile) and c.1204T>C (Tyr402His) single-nucleotide polymorphisms (SNPs) in CFH. No genetic abnormalities were revealed in complement factor B, complement factor I, CFH-related protein 5, membrane cofactor protein, or thrombomodulin genes. Approval from the Research Ethics Committee of the Faculty of Medicine of University of Miyazaki and Institutional Review board of University of Tokyo and written informed consent from the patient were obtained to perform the genetic analysis.

## 3 Discussion

The present case appears to have been one of secondary TMA mediated by coagulopathy. In one of several reports of TMA cases, Sakamaki et al^[7]^ reported a case of sepsis-induced DIC complicated with TMA that was successfully treated via plasma exchange, with a hypothesized cause of TMA progression due to sepsis-induced DIC. Furthermore, Booth et al^[8]^ have reported 10 of 31 cases from the Oklahoma Thrombotic Thrombocytopenic Purpura-Hemolytic Uremic Syndrome registry, in which the patients presented with clinical signs of thrombotic thrombocytopenic purpura (TTP) after systemic infection, developing DIC. In the present case, the elevated blood endotoxin levels led us to conclude that the patient had overt DIC triggered by a gram-negative bacterial infection, which induced TMA. Similarly, there are reports of secondary TMA caused by pregnancy or other underlying diseases, such as autoimmune diseases. Some of these cases were successfully treated using eculizumab, although the patients did not have any known associated genetic mutations.^[9–12]^

The patient had elevated serum soluble C5b-9 levels before eculizumab treatment. It is reported that serum soluble C5b-9 levels are similar to those in plasma when the sample thawed at room temperature.^[13]^ Moreover, elevated levels of C5b-9 have been reported in cases of acquired TTP or sepsis-induced DIC, but not in specific atypical hemolytic uremic syndrome cases.^[14–16]^ However, the elevated level of soluble C5b-9 in our study was much higher than that reported in the case of septic DIC that was accompanied by complement component 3 reduction, indicating complement alternative pathway activation. Furthermore, because thrombocytopenia, hemolysis, and renal dysfunction subsided and the purpura severity reduced, after eculizumab treatment, the patient may have a state of not only DIC, but also defective complement control. Interestingly, the coagulation and complement systems mutually induce positive feedback.^[17]^ Thus, it is possible that the septic DIC triggered abnormal complement activation and led to complement-mediated TMA. In this patient, the pathological symptoms of TMA (renal dysfunction, hemolytic anemia, and reduced platelet count) did not improve after treatment for sepsis and resolution of DIC. Therefore, based on improvement of all symptoms after eculizumab treatment, it may be insufficient to treat sepsis and DIC when defective complement control is present, leading to TMA pathologies. Rather, it is more likely that controlling complement systems by eculizumab for secondary TMAs is a potentially beneficial treatment.

Genetic testing revealed compound heterozygous SNPs of c.184G>A (Val62Ile) and c.1204T>C (Tyr402His) in CFH. These SNPs are present in about 31% and 13%, respectively, of a healthy population.^[18,19]^ Although c.1204T>C (Tyr402His) has not been published as a risk factor for aHUS, it is located in the lesion of aHUS-related mutation hotspots.^[20]^ Genetic mutations are not detected in approximately 50% of aHUS cases^[21,22]^; therefore, further investigations clarifying the relation between aHUS pathogenesis and SNPs on complement or coagulation genes are needed.

When critically ill patients exhibit signs of TMAs, there is insufficient time to wait for genetic testing results. Gilbert et al^[23]^ have reported 2 cases in which eculizumab improved the condition of genetically normal infants with secondary TMA following *S pneumoniae* infection without known genetic mutations of aHUS. Other reports recommend starting eculizumab treatment in patients with TMA, after excluding Shiga toxin-producing *E coli*-induced hemolytic uremic syndrome (STEC-HUS) and TTP, as this allows clinicians to initiate treatment without waiting for genetic testing results for complement regulatory factors.^[2–5]^ As eculizumab was effective for resolving complement-mediated TMA induced by DIC, we believe that patients who exhibit TMA findings might benefit from eculizumab treatment after STEC-HUS and TTP are excluded, even in cases of sepsis-induced TMA.

However, treatment using eculizumab is expensive, with an annual cost of approximately US $500,000 for an adult patient in Japan. Furthermore, few studies have evaluated the cost-effectiveness of eculizumab and its outcomes, such as reductions in the duration of renal replacement therapy, the number of plasma therapies, and/or the duration of hospital stays. Moreover, there is a risk of meningococcal infection with eculizumab treatment. There is also no standardized effective prophylaxis against the development of meningococcal infection during eculizumab treatment. Thus, further research and prospective studies are needed to support the wide-spread use of eculizumab for secondary TMA, such as sepsis-induced TMA accompanied by coagulopathy.

We described a case of complement-mediated TMA complicated with overt DIC, in which we were unable to achieve clinical improvement by treatment for only sepsis and DIC, but for which we achieved resolution of TMA using eculizumab. When TMA findings, such as reduced platelet count, hemolytic anemia, and renal dysfunction, are complicated with DIC, it may be that the complications arise due to crosstalk between the complement and coagulation systems. Those TMAs might not be controllable by treatments for only underlying pathologies (e.g., sepsis). Therefore, we suggest treating the underlying pathologies first; the present case demonstrated that persistent TMA may be resolved with eculizumab use. Further studies are needed to support more widespread use of eculizumab for secondary TMAs.

## Acknowledgments

The authors would like to thank Hideki Kato, MD, PhD, for assistance with genetic testing and Hideaki Imamura, MD, for helping with C5b-9 testing in the present case.
